# Comparative Impact of an Optimized PK/PD Target Attainment of Piperacillin-Tazobactam vs. Meropenem on the Trend over Time of SOFA Score and Inflammatory Biomarkers in Critically Ill Patients Receiving Continuous Infusion Monotherapy for Treating Documented Gram-Negative BSIs and/or VAP

**DOI:** 10.3390/antibiotics13040296

**Published:** 2024-03-25

**Authors:** Milo Gatti, Matteo Rinaldi, Tommaso Tonetti, Antonio Siniscalchi, Pierluigi Viale, Federico Pea

**Affiliations:** 1 Department of Medical and Surgical Sciences, Alma Mater Studiorum University of Bologna, 40138 Bologna, Italy; milo.gatti2@unibo.it (M.G.); matteo.rinaldi23@unibo.it (M.R.); tommaso.tonetti@unibo.it (T.T.); pierluigi.viale@unibo.it (P.V.); 2 Clinical Pharmacology Unit, Department for Integrated Infectious Risk Management, IRCCS Azienda Ospedaliero-Universitaria of Bologna, 40138 Bologna, Italy; 3 Infectious Disease Unit, Department for Integrated Infectious Risk Management, IRCCS Azienda Ospedaliero-Universitaria of Bologna, 40138 Bologna, Italy; 4 Division of Anesthesiology, Department of Anesthesia and Intensive Care, IRCCS Azienda Ospedaliero-Universitaria di Bologna, 40138 Bologna, Italy; 5 Anesthesia and Intensive Care Medicine, IRCCS Azienda Ospedaliero-Universitaria di Bologna, 40138 Bologna, Italy; antonio.siniscalchi@aosp.bo.it

**Keywords:** piperacillin-tazobactam, meropenem, continuous infusion, critically ill patients, gram-negative infections, SOFA score, inflammatory biomarkers, clinical outcome

## Abstract

(1) Background: The advantage of using carbapenems over beta-lactam/beta-lactamase inhibitor combinations in critically ill septic patients still remains a debated issue. We aimed to assess the comparative impact of an optimized pharmacokinetic/pharmacodynamic (PK/PD) target attainment of piperacillin-tazobactam vs. meropenem on the trend over time of both Sequential Organ Failure Assessment (SOFA) score and inflammatory biomarkers in critically ill patients receiving continuous infusion (CI) monotherapy with piperacillin-tazobactam or meropenem for treating documented Gram-negative bloodstream infections (BSI) and/or ventilator-associated pneumonia (VAP). (2) Methods: We performed a retrospective observational study comparing critically ill patients receiving targeted treatment with CI meropenem monotherapy for documented Gram-negative BSIs or VAP with a historical cohort of critical patients receiving CI piperacillin-tazobactam monotherapy. Patients included in the two groups were admitted to the general and post-transplant intensive care unit in the period July 2021–September 2023 and fulfilled the same inclusion criteria. The delta values of the SOFA score between the baseline of meropenem or piperacillin-tazobactam treatment and those at 48-h (delta 48-h SOFA score) or at 7-days (delta 7-days SOFA) were selected as primary outcomes. Delta 48-h and 7-days C-reactive protein (CRP) and procalcitonin (PCT), microbiological eradication, resistance occurrence, clinical cure, multi-drug resistant colonization at 90-day, ICU, and 30-day mortality rate were selected as secondary outcomes. Univariate analysis comparing primary and secondary outcomes between critically ill patients receiving CI monotherapy with piperacillin-tazobactam vs. meropenem was carried out. (3) Results: Overall, 32 critically ill patients receiving CI meropenem monotherapy were compared with a historical cohort of 43 cases receiving CI piperacillin-tazobactam monotherapy. No significant differences in terms of demographics and clinical features emerged at baseline between the two groups. Optimal PK/PD target was attained in 83.7% and 100.0% of patients receiving piperacillin-tazobactam and meropenem, respectively. No significant differences were observed between groups in terms of median values of delta 48-h SOFA (0 points vs. 1 point; *p* = 0.89) and median delta 7-days SOFA (2 points vs. 1 point; *p* = 0.43). Similarly, no significant differences were found between patients receiving piperacillin-tazobactam vs. meropenem for any of the secondary outcomes. (4) Conclusion: Our findings may support the contention that in critically ill patients with documented Gram-negative BSIs and/or VAP, the decreases in the SOFA score and in the inflammatory biomarkers serum levels achievable with CI piperacillin-tazobactam monotherapy at 48-h and at 7-days may be of similar extent and as effective as to those achievable with CI meropenem monotherapy provided that optimization on real-time by means of a TDM-based expert clinical pharmacological advice program is granted.

## 1. Introduction

Bacterial infections cause most cases of sepsis, which is a leading cause of morbidity and mortality in the intensive care unit (ICU) [1,2,3]. Bloodstream infections (BSIs) and ventilator-associated pneumonia (VAP) may account for a large part of these infections in critically ill patients [3,4]. About two-thirds of the bacterial infections in critically ill septic patients are caused by *Enterobacterales* and/or by *Pseudomonas aeruginosa* [3,4,5].

Piperacillin-tazobactam, a traditional beta-lactam/beta-lactamase inhibitor combination (BL/BLIc), and meropenem, a carbapenem, represent the cornerstone of empirical and targeted therapy of Gram-negative BSIs and VAP in the critically ill patients [6,7,8,9,10,11,12]. Several studies showed no significant difference between piperacillin-tazobactam or meropenem monotherapy in the clinical outcome of patients with severe Gram-negative bacterial infections, including those caused by extended-spectrum beta-lactamase (EBSL)-producing *Enterobacterales* [13,14,15,16,17,18,19,20,21,22,23,24,25,26,27].

Currently, real-time therapeutic drug monitoring (TDM)-guided dosage optimization of beta-lactam treatment is gaining more and more relevance and is considered the only safe and effective way for granting optimal pharmacokinetic/pharmacodynamic (PK/PD) target attainment among critically ill patients [28,29]. Several studies showed that attaining an aggressive PK/PD target of 100%T_>4xMIC_ was associated with favorable microbiological/clinical outcomes of beta-lactam monotherapy [30,31,32,33,34].

However, the advantages of an optimized treatment with meropenem monotherapy over piperacillin/tazobactam for severe documented Gram-negative bacterial infections still remain to be investigated.

Valuable tools for addressing this issue could be assessing the trend over time of both the Sequential Organ Failure Assessment (SOFA) score and the inflammatory biomarkers, like C-reactive protein (C-RP) and procalcitonin (PCT) [35,36,37,38]. On the one hand, the SOFA score may be informative about the impact that antibiotic treatment may have on organ dysfunction, which is a primary focus considering that sepsis may be frequently associated with organ failure [35,36,39]. Thanks to SOFA having a scalar nature, the number of patients needed for testing the impact of treatment efficacy on SOFA score would be smaller than that required for testing treatment efficacy on mortality. Consequently, an ever-growing number of studies have adopted SOFA score as a primary or secondary endpoint [36]. On the other hand, decreases over time of inflammatory biomarkers like C-RP and PCT are considered valuable indicators for establishing when stopping antibiotic therapy [6,40]. Several studies and meta-analyses showed that adopting an inflammatory biomarker-guided approach for stopping antibiotic therapy in critically ill patients was significantly associated with better clinical outcomes, including an improvement in mortality rate [37,38,41,42,43,44,45,46,47,48,49,50].

The aim of this study was to assess the comparative impact of an optimized PK/PD target attainment of meropenem vs. piperacillin-tazobactam on the trend over time of both SOFA score and inflammatory biomarkers in critically ill patients receiving continuous infusion (CI) beta-lactam monotherapy for treating documented Gram-negative BSI and/or VAP.

## 2. Results

**Figure 1:**
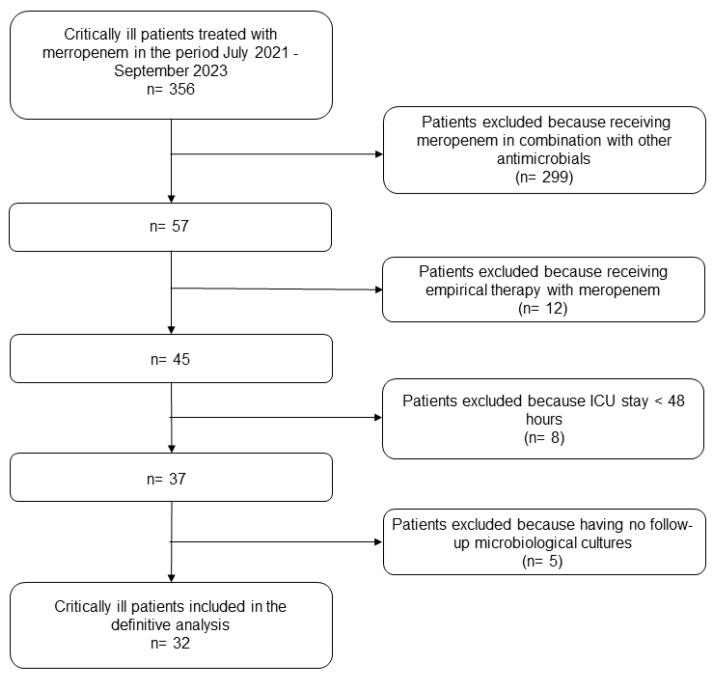
Flowchart of patient inclusion and exclusion criteria for meropenem group. In regard to inclusion and exclusion criteria for piperacillin-tazobactam group refers to [51].

Overall, 32 critically ill patients were included in the meropenem cohort (Figure 1) and compared with 43 patients belonging to the historical piperacillin-tazobactam cohort (Table 1) [51].

No significant difference in terms of demographics and clinical features emerged between the two cohorts. Specifically, the two cohorts were comparable in terms of need for mechanical ventilation (81.4% vs. 75.0%; *p* = 0.51), vasopressors support (62.8% vs. 62.5%; *p* = 0.98), continuous renal replacement therapy (CRRT) application (25.6% vs. 31.3%; *p* = 0.59), occurrence of augmented renal clearance (ARC; 7.0% vs. 9.4%; *p* = 0.99) median baseline values of SOFA score (8 points vs. 9 points; *p* = 0.56), CRP (14.9 mg/dL vs. 16.1 mg/dL), and of PCT serum levels (4.7 vs. 8.7 ng/mL).

VAP was more represented in the piperacillin-tazobactam cohort than in the meropenem cohort (37.2% vs. 15.6%; *p* = 0.04), but when merging VAP with bacteraemic VAP no difference was found (44.2% vs. 34.4%; *p* = 0.39). *Escherichia coli* (37.5% vs. 17.9%; *p* = 0.046) and *Pseudomonas aeruginosa* (29.0% vs. 10.3%; *p* = 0.04) were the predominant pathogens in the piperacillin-tazobactam cohort, whereas *Klebsiella pneumoniae* was the prevalent one in the meropenem cohort (35.9% vs. 12.5%; *p* = 0.01). ESBL-producing (14.6% vs. 30.8%; *p* = 0.07) and AmpC-producing *Enterobacterales* (6.3% vs. 10.3%; *p* = 0.70) were equally represented in the two cohorts.

Optimal PK/PD target was attained in 83.7% and 100.0% of patients in the piperacillin-tazobactam and the meropenem cohort, respectively (*p* = 0.06). Suboptimal joint PK/PD target attainment was documented only in one patient belonging to the piperacillin-tazobactam cohort. The number of instances of TDM-guided dosing adjustments was similar in the two cohorts.

Univariate analysis evaluating the primary and secondary outcomes in critically ill patients treated with piperacillin-tazobactam vs. meropenem is reported in Table 2.

**Figure 2:**
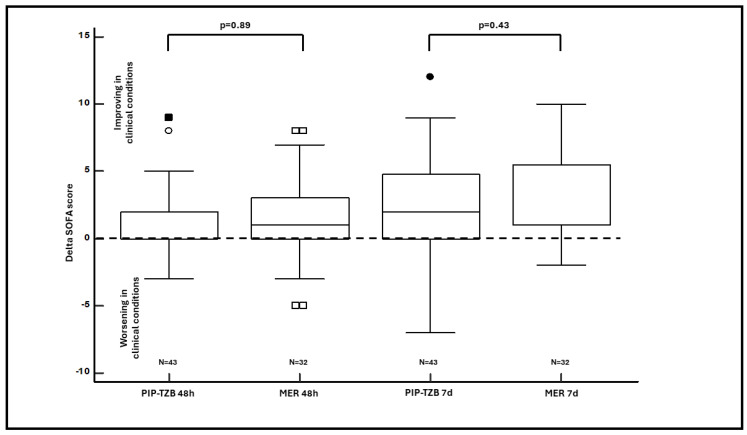
Box-and-whisker plot of the median delta SOFA score at 48-h and at 7-days in piperacillin-tazobactam vs. meropenem group. The dotted line indicates a delta SOFA score equal to 0. Dots and squares represent the outliers. MER: meropenem; PIP-TZB; piperacillin-tazobactam; SOFA: Sequential Organ Failure Assessment.

In regard to the primary outcome (Figure 2), no significant difference, neither in terms of median delta 48-h SOFA (0 points vs. 1 point; *p* = 0.89) nor of median delta 7-days SOFA (2 points vs. 1 point; *p* = 0.43), were observed between the two groups.

**Figure 3:**
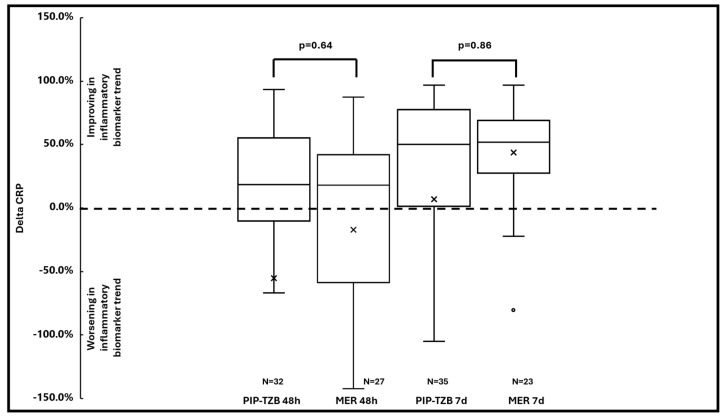
Box-and-whisker plot of the median delta CRP at 48-h and at 7-days in piperacillin-tazobactam vs. meropenem group. Dot and x represent the outliers. The dotted line indicates a delta CRP equal to 0.0%. CRP: C-reactive protein; MER: meropenem; PIP-TZB; piperacillin-tazobactam.

**Figure 4:**
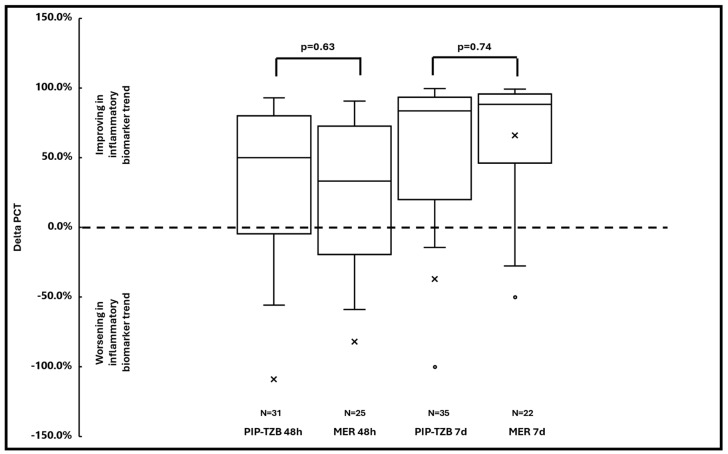
Box-and-whisker plot of the median delta PCT at 48-h and at 7-days in piperacillin-tazobactam vs. meropenem group. Dot and x represent the outliers. The dotted line indicates a delta PCT equal to 0.0%. MER: meropenem; PCT: procalcitonin; PIP-TZB; piperacillin-tazobactam.

In regard to the secondary outcomes, no significant difference, neither in the median delta CRP serum levels at 48-h (18.3% vs. 18.0%; *p* = 0.64) and at 7-days (50.3% vs. 51.8%; *p* = 0.86) (Figure 3), nor in the median delta of PCT serum levels at 48-h (50.0% vs. 33.3%; *p* = 0.63) and at 7-days (83.5% vs. 88.1%; *p* = 0.74; Figure 4), were found between the two cohorts.

Likewise, no significant difference, in terms of microbiological eradication rate (74.4% vs. 84.4%; *p* = 0.30), resistance occurrence (7.0% vs. 6.9%; *p* = 0.99), clinical cure (67.4% vs. 71.9%; *p* = 0.68), MDR colonization at 90-days (9.3% vs. 15.6%; *p* = 0.48), ICU mortality rate (9.3% vs. 18.8%; *p* = 0.31), and 30-day mortality rate (14.0% vs. 21.9%; *p* = 0.37), emerged between the two groups.

Univariate analysis evaluating SOFA subscores at 48-h and at 7-days for each of the six items in the two cohorts is reported in Table 3.

No significant difference for any of the six items composing the SOFA subscores was found between the two groups, neither at 48-h nor at 7-days.

## 3. Discussion

To the best of our knowledge, this is the first study that evaluated the clinical impact of implementing an optimized PK/PD target attainment of CI monotherapy with piperacillin-tazobactam vs. meropenem on the decrease in the SOFA score and in the inflammatory biomarkers serum levels achievable at 48-h and at 7-days in critically ill patients affected by documented Gram-negative BSIs and/or VAP.

The findings showed no significant difference in terms of decrease in SOFA score at 48-**h** and at 7-days between critically ill patients receiving TDM-guided optimized therapy with CI piperacillin-tazobactam vs. meropenem. Likewise, no significant advantages were observed in the meropenem cohort over the piperacillin-tazobactam cohort in time to decrease inflammatory biomarkers serum levels. Notably, similar microbiological eradication rates and clinical cure rates were also observed.

Overall, these findings could support the contention that using piperacillin-tazobactam could be a feasible carbapenem-sparing strategy in challenging scenarios of documented Gram-negative infections provided that a real time TDM-guided ECPA program would be available for optimizing PK/PD target attainment.

Previous studies reported conflicting results in terms of the clinical efficacy of piperacillin-tazobactam vs. meropenem in treating critically ill patients with sepsis and/or septic shock [52,53]. A meta-analysis of thirty-one randomized controlled trials including different BL/BLICs compared to carbapenems in septic patients found no difference in regard to mortality rate, clinical failure, microbiological failure, and bacterial superinfections [53]. Conversely, a recent randomized controlled trial including 622 patients receiving meropenem vs. 622 receiving piperacillin-tazobactam for treating sepsis and/or septic shock reported a significantly better improvement in hospital duration stay, respiratory and renal SOFA scores, and intervention-free days from renal replacement therapy in the meropenem arm [52]. However, most of these studies should be interpreted cautiously because some major biases, namely the absence of documented Gram-negative infections, and/or use of combination therapy, could have affected the proper evaluation of treatment impact on clinical efficacy and SOFA score trend.

The supposed advantage of using carbapenems over BL/BLICs in critically ill septic patients still remains a debated issue. In this scenario, our analysis could provide some clues for supporting the fact that a targeted monotherapy with piperacillin-tazobactam may be non-inferior vs. meropenem in terms of impact on recovery from multiorgan failure, decrease in inflammatory biomarkers trend, and clinical outcome when aggressive PK/PD targets are attained. Indeed, it is noteworthy that different studies reported that attaining an aggressive beta-lactam PK/PD target of 100%*f*T_>4-8xMIC_ among critically ill patients was associated with both maximization of clinical efficacy and suppression of resistance development [30,31,32,33,34,54,55,56,57,58]. Furthermore, failure in attaining aggressive PK/PD targets with beta-lactams was independently associated with a significantly higher risk of microbiological failure [31,51]. Unfortunately, the relevant pathophysiological alterations occurring in critically ill patients may affect the likelihood of attaining aggressive beta-lactam PK/PD targets [5,29,59,60,61,62]. Consequently, implementing a real-time TDM-guided ECPA program could be helpful in addressing this issue by providing proper dosing adjustments for promptly attaining aggressive PK/PD target [28], as witnessed by our findings. Indeed, it should be mentioned that approximately 80% of the clinical isolates yielded in the meropenem group were very susceptible, namely with an MIC of 0.12 mg/L. This favored the attainment of very high C_ss_/MIC ratios of meropenem in the vast majority of cases, despite the fact that the adopted CI meropenem dosing regimens were low. We recognize that in this scenario implementing a TDM-guided approach could have a limited impact in terms of clinical outcome, but it should not be overlooked that this approach could be very helpful also in minimizing unnecessary meropenem overexposure and therefore in containing as much as possible the carbapenem selective pressure.

It is worth mentioning that attaining aggressive PK/PD targets was impactful in ameliorating sepsis-related organ dysfunction in both of the cohorts, as shown by the relevant magnitude improvement of the delta SOFA score at 7-days. This is a further element PK/PD targets as an effective antimicrobial strategy for minimizing the overuse of carbapenems also in challenging settings. This is in agreement with recent position papers and international guidelines strongly recommending the adoption of a routinely TDM-guided approach for optimizing beta-lactam PK/PD target attainment in critically ill patients [63,64]. Conversely, our findings are different from what was previously observed in two other studies [65,66]. Specifically, Alshaer et al. in treating ICU patients with Gram-negative pneumonia and/or BSI with cefepime adopted a PK/PD target much less aggressive than ours, namely 100%*f*T_>MIC_, and reported that target attainment was associated with only a negligible decrease in vasopressors requirement and with no impact on SOFA score change [65]. Hagel et al. in a randomized, multicenter, controlled trial of patients with sepsis or septic shock randomly assigned 1:1 to receive TDM-guided or non-TDM-guided CI piperacillin/tazobactam found that the TDM-guided approach was not associated with improvement in mean SOFA score at day 10 [66]. However, it should be mentioned that in this study documented Gram-negative infections accounted for less than half of cases, clinical isolates were very susceptible to piperacillin/tazobactam and combo therapy was allowed [66].

Limitations of our study have to be recognized. The retrospective monocentric design, the limited sample size, and the availability of inflammatory biomarker serum levels at the predefined time points in not all but around 70–75% of cases should be acknowledged. We recognize that performing accurate sample size calculations would have added more value to the study for establishing the statistical power. Unfortunately, we did not have a benchmark for sample size calculation since, to the best of our knowledge, this was the first study exploring the comparative impact of such an intervention, namely an optimized PK/PD target attainment of piperacillin-tazobactam vs. meropenem monotherapy, on delta SOFA score, delta CR-P, and delta PCT over time. However, we could argue that the total number of patients included in our study (75) could suffice for inferring reliable conclusions, considering that it is of similar magnitude to the median (IQR) number of patients [64 (40–147 patients)] included in a recent review of 87 RCTs exploring a suchlike field, namely the clinical impact of several interventions on SOFA score variations over time in septic and/or septic shock patients [36]. Interestingly, in the hypothesis of aiming to detect a difference of 1 point in the mean 7-days delta SOFA score, namely a value that in [36] was associated with a mortality odds ratio of 2.0, we estimated that a total of 70 patients (35 in each group), similar to our cohort, could have been appropriate with a type-I error rate of 5% and a power level of 80%. Conversely, including only critically ill patients receiving piperacillin-tazobactam or meropenem monotherapy for treating documented Gram-negative BSIs and/or VAP may represent a point of strength of our study. This may have minimized as much as possible any potential confounding factors in evaluating SOFA score and inflammatory biomarkers trends in the two cohorts.

## 4. Materials and Methods

### 4.1. Study Design and Inclusion Criteria

This retrospective observational study compared a cohort of critically ill patients receiving targeted CI meropenem monotherapy (meropenem cohort) optimized by means of a real-time TDM-guided expert clinical pharmacological advice (ECPA) program for documented Gram-negative BSIs or VAP with a historical cohort of critical patients receiving CI piperacillin-tazobactam monotherapy (piperacillin-tazobactam cohort) for the same indication and optimized by means of the same approach [51]. Inclusion criteria for the meropenem cohort were the same adopted for the historical piperacillin-tazobactam cohort [51], namely: (a) admission to the general or the post-transplant ICU of the IRCCS Azienda Ospedaliero-Universitaria of Bologna, Italy in the period between 1 July 2021 and 15 September 2023; (b) documented Gram-negative BSIs or VAP with available minimum inhibitory concentration (MIC) value for meropenem or piperacillin-tazobactam; (c) targeted antimicrobial monotherapy therapy with CI meropenem or piperacillin-tazobactam for at least 48-h during ICU stays; (d) real-time TDM-guided approach for optimizing meropenem or piperacillin-tazobactam PK/PD target attainment during ICU stay; (e) no change in antimicrobial therapy during the treatment course; (f) no implementation of compassionate care, discharge, or death in the first 48-h after ICU admission.

### 4.2. Data Collection

Demographic (age, sex, weight, height, body mass index [BMI]) and clinical/laboratory data (underlying disease leading to ICU admission, requirement for mechanical ventilation, for vasopressors, and for CRRT, occurrence of ARC, SOFA score at the start of the treatment course, baseline PCT and CRP serum levels) were collected for each patient. Furthermore, microbiological (type/site of infection, Gram-negative isolates with relative MIC values) and antibiotic treatment data (dosing at baseline, steady-state concentrations [C_ss_] at first TDM-guided ECPA, average meropenem or piperacillin-tazobactam C_ss_ during treatment course in patients in which more than one TDM-guided ECPA was performed, overall number of ECPAs, recommended dosing adjustments at first and at subsequent ECPAs) were also retrieved.

CRRT application was defined as the implementation of continuous venovenous hemofiltration (CVVH), hemodialysis (CVVHD), or hemodiafiltration (CVVHDF) for at least 24-h during antibiotic treatment course [67].

ARC was defined as an estimated (based on the CDK-EPI formula) or measured (according to 24-h urine collection) creatinine clearance over 130 mL/min and 120 mL/min in males and females, respectively [68,69].

BSI and VAP were defined according to the Centers for Disease Control and Prevention (CDC) criteria [70], as previously reported [51].

The MIC values for meropenem against Gram-negative isolates were determined by means of a semi-automated broth microdilution method (Microscan Beckman NMDRM1), and interpreted according to The European Committee on Antimicrobial Susceptibility Testing (EUCAST) clinical breakpoints [71].

### 4.3. Outcome Definition

The delta values between the SOFA score at baseline of meropenem or piperacillin-tazobactam treatment and those at 48-h (delta 48-h SOFA score) or at 7-days (delta 7-days SOFA score) were selected as primary outcomes. Delta 48-h and 7-days SOFA scores were calculated as follows:

delta 48-h SOFA score = baseline SOFA score value—48-h SOFA score value;

delta 7-days SOFA score = baseline SOFA score value—7-days SOFA score value

Whenever discharge from the ICU or death occurred before day 7, the delta 7-days SOFA score was assessed by assuming the last day on which SOFA score was assessable. The Delta 48-h and 7-days values were assessed also for each of the 6 items composing the SOFA score assessment (namely cardiovascular, respiratory, coagulation, renal, hepatic, and neurological) and were defined as Delta 48-h and 7-days SOFA subscores [39].

Delta 48-h and 7-days for CRP and PCT, microbiological eradication, resistance occurrence, clinical cure, multi-drug resistant (MDR) colonization at 90-day, ICU, and 30-day mortality rates were selected as secondary outcomes.

The delta 48-h and 7-days for CRP were defined as the difference between the CRP serum level at baseline of antibiotic treatment and those at 48-h and at 7-days, respectively. Similarly, the delta 48-h and 7-days for PCT were defined as the difference between the PCT serum levels at baseline antibiotic treatment and those at 48-h and at 7-days, respectively. Whenever discharge from ICU or death occurred before day 7, delta 7-days for CRP and/or PCT were assessed by assuming the last day in which CRP and/or PCT serum levels were assessable.

Microbiological eradication was defined as the absence of the index Gram-negative pathogens in at least two follow-up microbiological cultures collected from the primary site of infection (blood culture or bronchoalveolar lavage in case of BSI or VAP, respectively) [72]. Resistance occurrence was defined as the isolation of Gram-negative pathogens at the follow-up microbiological cultures with an MIC value for piperacillin-tazobactam or meropenem above the EUCAST susceptibility clinical breakpoint.

Clinical cure was defined as the complete resolution of signs and symptoms related to the infection coupled with documented microbiological eradication and absence of relapse at 30-day follow-up [73].

MDR colonization at 90-days was defined as the finding at surveillance rectal swabs or urine culture of a novel difficult-to-treat resistant (DTR) Gram-negative pathogen in the absence of signs or symptoms of infection in the subsequent 90-days after starting piperacillin-tazobactam or meropenem treatment course.

### 4.4. Antibiotic Dosing Regimens, Sampling Procedure, Definition of Optimal PK/PD Target Attainment, and Procedure for Optimizing PK/PD Target Attainment

Targeted monotherapy with piperacillin-tazobactam or meropenem was always started with a loading dose followed by a CI maintenance dose with timing for solutions reconstitution depending on the selected agent, as previously detailed according to stability in aqueous solution [74].

First TDM was carried out in steady-state conditions after at least 24-h and total antibiotic plasma concentrations were assessed by means of a validated liquid chromatography-tandem mass spectrometry method [31]. Subsequent TDM-guided ECPA reassessments were performed every 48–72-h during the ICU stay. Free (*f*) meropenem, piperacillin, and tazobactam C_ss_ were calculated according to the plasma protein binding rate reported in the literature, as previously detailed [74].

In regard to meropenem, PK/PD target attainment was defined as optimal, quasi-optimal, and suboptimal depending on the *f*C_ss_/MIC ratio being >4, 1–4, and <1, respectively. In regard to piperacillin-tazobactam, a joint PK/PD target was adopted, as previously defined [51]. Specifically, the joint PK/PD target was defined as optimal when both the piperacillin *f*C_ss_/MIC ratio was >4 and the tazobactam *f*C_ss_/C_T_ ratio was >1 (where C_T_ corresponded to the fixed tazobactam target concentration used by the EUCAST for the in vitro standard susceptibility testing, namely, 4 mg/L). Quasi-optimal or suboptimal PK/PD targets were defined according to the fact that only one or none of the two thresholds were attained, respectively [51].

### 4.5. Statistical Analysis

Continuous data were presented as median and interquartile range (IQR), whereas categorical variables were expressed as counts or percentages. Univariate analysis was implemented for comparing primary and secondary outcomes in critically ill patients receiving piperacillin-tazobactam vs. meropenem monotherapy by means of the Fisher’s exact test or the chi-squared test for categorical variables, or the Mann–Whitney U test for continuous variables. A *p*-value < 0.05 was selected for defining statistical significance. Statistical analyses were carried out by means of the MedCalc statistical software (Version 19.6.1, Ostend, Belgium).

## 5. Conclusions

In conclusion, our findings may support the contention that in critically ill patients with documented Gram-negative BSIs and/or VAP, the decreases in the SOFA score and in the inflammatory biomarkers serum levels achievable with CI piperacillin-tazobactam monotherapy at 48-h and at 7-days may be of similar extent and as effective as to those achievable with CI meropenem monotherapy provided that optimization on real-time by means of an TDM-based ECPA program is granted. Larger prospective studies are warranted to confirm our hypothesis.

## Figures and Tables

**Table 1:** Comparison between demographics and clinical features of ICU patients receiving CI monotherapy with piperacillin-tazobactam vs. meropenem for documented Gram-negative BSIs and/or VAP.

Demographics and Clinical Variables	Piperacillin-Tazobactam (*n* = 43)	Meropenem (*n* = 32)	*p* Value
*Patient demographics*			
Age (years) (median (IQR))	69 (57–74)	71.5 (61.25–76.25)	0.28
Gender (male/female) (*n* (%))	25/18 (58.1/41.9)	24/8 (75.0/25.0)	0.13
Body weight (Kg) (median (IQR))	80 (65–90)	80 (70–90)	0.52
Body mass index (Kg/m^2^) (median (IQR))	26.1 (23.1–29.4)	27.6 (24.2–32.5)	0.31
*Severity of clinical conditions*			
Mechanical ventilation (*n* (%))	35 (81.4)	24 (75.0)	0.51
Vasopressors (*n* (%))	27 (62.8)	20 (62.5)	0.98
Continuous renal replacement therapy (*n* (%))	11 (25.6)	10 (31.3)	0.59
Augmented renal clearance (*n* (%))	3 (7.0)	3 (9.4)	0.99
Baseline SOFA score (median (IQR))	8 (4–11)	9 (5.75–13)	0.56
Baseline serum PCT levels (median (IQR))	4.7 (0.6–34.0)	8.7 (2.0–58.8)	0.14
Baseline serum CRP levels (median (IQR))	14.9 (7.1–23.3)	16.1 (9.1–26.7)	0.33
*Site of infection* (*n* (%))			
BSI	24 (55.8)	21 (65.6)	0.39
VAP	16 (37.2)	5 (15.6)	**0.04**
VAP + BSI	3 (7.0)	6 (18.8)	0.16
*Gram-negative clinical isolates*^a^ (*n* (%))			
*Escherichia coli*	18 (37.5)	7 (17.9)	**0.046**
*Pseudomonas aeruginosa*	14 (29.0)	4 (10.3)	**0.04**
*Klebsiella pneumoniae*	6 (12.5)	14 (35.9)	**0.01**
*Klebsiella aerogenes*	2 (4.2)	3 (7.7)	0.65
*Proteus mirabilis*	2 (4.2)	1 (2.6)	0.99
*Proteus vulgaris*	2 (4.2)	0 (0.0)	0.50
*Serratia marcescens*	1 (2.1)	2 (5.1)	0.58
*Citrobacter koseri*	1 (2.1)	0 (0.0)	0.99
*Citrobacter braakii*	1 (2.1)	0 (0.0)	0.99
*Klebsiella oxytoca*	1 (2.1)	2 (5.1)	0.58
*Enterobacter cloacae*	0 (0.0)	2 (5.1)	0.20
*Enterobacter bugadensis*	0 (0.0)	1 (2.6)	0.45
*Morganella morganii*	0 (0.0)	1 (2.6)	0.45
*Acinetobacter baumannii*	0 (0.0)	1 (2.6)	0.45
*Hafnia alvei*	0 (0.0)	1 (2.6)	0.45
ESBL-producing *Enterobacterales*	7 (14.6)	12 (30.8)	0.07
AmpC-producing *Enterobacterales*	3 (6.3)	4 (10.3)	0.70
*Beta-lactam treatment*			
Daily dose (mg) (median (IQR))	18 g/day (13.5–18 g/day)	2 g/day (1.5–4 g/day)	
Piperacillin/Meropenem *f*C_ss_ (mg/L) (median (IQR))	54.6 (41.0–91.2)	14.9 (10.5–24.8)	
Tazobactam *f*C_ss_ (mg/L) (median (IQR))	7.2 (4.6–11.6)	-	
Piperacillin/Meropenem *f*C_ss_/MIC ratio (median (IQR))	7.6 (4.8–13.0)	92.3 (20.3–166.5)	
Tazobactam *f*C_ss_/C_T_ ratio (median (IQR))	1.8 (1.2–2.9)	-	
*PK/PD target attainment*			
Overall optimal joint PK/PD target (*n* (%))	36 (83.7)	32 (100.0)	
Overall quasi-optimal joint PK/PD target (*n* (%))	6 (14.0)	0 (0.0)	0.06
Overall suboptimal joint PK/PD target (*n* (%))	1 (2.3)	0 (0.0)	
*ECPA program*			
Overall TDM-based ECPAs	93	80	
N. of TDM-based ECPA per treatment course (median (IQR))	2 (1–2.5)	2 (1–3.25)	0.48
N. of dosage confirmations at first TDM assessment (*n* (%))	15 (34.9)	7 (21.9)	0.22
N. of dosage increases at first TDM assessment (*n* (%))	1 (2.3)	0 (0.0)	0.99
N. of dosage decreases at first TDM assessment (*n* (%))	27 (62.8)	25 (78.1)	0.16
Overall n. of dosage confirmations (*n* (%))	49 (52.7)	38 (47.5)	0.50
Overall n. of dosage increases (*n* (%))	5 (5.4)	4 (5.0)	0.99
Overall n. of dosage decreases (*n* (%))	39 (41.9)	38 (47.5)	0.46

BSI: bloodstream infection; CRP: C-reactive protein; ECPA: expert clinical pharmacological advice; ESBL: extended-spectrum beta-lactamase; *f*C_ss_: free steady-state concentrations; *f*C_T_: free threshold concentrations; ICU: intensive care unit; IQR: interquartile range; MIC: minimum inhibitory concentration; PCT: procalcitonin; PK/PD: pharmacokinetic/pharmacodynamic; SOFA: sequential organ failure assessment; TDM: therapeutic drug monitoring; VAP: ventilator-associated pneumonia. ^a^ Overall, 48 different Gram-negative pathogens were identified in the 43 ICU patients receiving CI piperacillin-tazobactam vs. 39 different Gram-negative pathogens identified in the 32 ICU patients treated with CI meropenem.

**Table 2:** Univariate analysis assessing primary and secondary outcomes in critically ill patients treated with piperacillin-tazobactam vs. meropenem.

Outcome	Piperacillin-Tazobactam (*n* = 43)	Meropenem (*n* = 32)	*p* Value
*Primary outcomes*			
Delta 48-h SOFA (median (IQR))	0 (0–2)	1 (0–3)	0.89
Delta 7-days SOFA (median (IQR))	2 (0–4.5)	1 (1–5.25)	0.43
*Secondary outcomes*			
Delta 48-h CRP (median (IQR)) *	18.3% (−9.2–43.2%)	18.0% (−47.9–41.1%)	0.64
Delta 7-days CRP (median (IQR)) **	50.3% (14.4–72.3%)	51.8% (27.8–67.9%)	0.86
Delta 48-h PCT (median (IQR)) ***	50.0% (2.6–77.6%)	33.3% (−11.5–70.9%)	0.63
Delta 7-days PCT (median (IQR)) ****	83.5% (35.0–93.2%)	88.1% (52.3–94.7%)	0.74
Microbiological eradication (*n* (%))	32 (74.4)	27 (84.4)	0.30
Resistance occurrence (*n* (%))	3 (7.0)	2 (6.3)	0.99
Clinical cure (*n* (%))	29 (67.4)	23 (71.9)	0.68
90-days MDR colonization (*n* (%))	4 (9.3)	5 (15.6)	0.48
ICU mortality (*n* (%))	4 (9.3)	6 (18.8)	0.31
30-day mortality (*n* (%))	6 (14.0)	7 (21.9)	0.37

CRP: C-reactive protein; ICU: intensive care unit; IQR: interquartile range; MDR: multidrug-resistant; PCT: procalcitonin; SOFA: sequential organ failure assessment. * Delta 48-h CRP was available in 32 out of 43 patients in piperacillin-tazobactam arm, and in 27 out of 32 patients in meropenem arm. ** Delta 7-days CRP was available in 35 out of 43 patients in piperacillin-tazobactam arm, and in 23 out of 32 patients in meropenem arm. *** Delta 48-h PCT was available in 31 out of 43 patients in piperacillin-tazobactam arm, and in 25 out of 32 patients in meropenem arm. **** Delta 7-days PCT was available in 35 out of 43 patients in piperacillin-tazobactam arm, and in 22 out of 32 patients in meropenem arm.

**Table 3:** Univariate analysis evaluating delta SOFA subscore values at 48-h and at 7-days in critically ill patients treated with piperacillin-tazobactam vs. meropenem.

Outcome	Piperacillin-Tazobactam (*n* = 43)	Meropenem (*n* = 32)	*p* Value
Delta 48-h cardiovascular SOFA subscore (median (IQR))	0 (0–1)	0 (0–1)	0.40
Delta 7-days cardiovascular SOFA subscore (median (IQR))	1 (0–4)	0 (0–4)	0.98
Delta 48-h respiratory SOFA subscore (median (IQR))	0 (0–1)	0 (0–1)	0.57
Delta 7-days respiratory SOFA subscore (median (IQR))	0 (0–1)	1 (0–1)	0.17
Delta 48-h coagulation SOFA subscore (median (IQR))	0 (−1–0)	0 (0–0)	0.16
Delta 7-days coagulation SOFA subscore (median (IQR))	0 (−0.5–0)	0 (0–0.25)	0.13
Delta 48-h renal SOFA subscore (median (IQR))	0 (0–0)	0 (−0.25–0)	0.46
Delta 7-days renal SOFA subscore (median (IQR))	0 (0–0.5)	0 (0–1)	0.78
Delta 48-h hepatic SOFA subscore (median (IQR))	0 (0–0)	0 (0–0)	0.49
Delta 7-days hepatic SOFA subscore (median (IQR))	0 (0–0)	0 (0–0.25)	0.61
Delta 48-h neurological SOFA subscore (median (IQR))	0 (0–0)	0 (0–0)	0.27
Delta 7-days neurological SOFA subscore (median (IQR))	0 (0–1)	0 (0–0)	0.55

IQR: interquartile range; SOFA: sequential organ failure assessment.

## Data Availability

The data presented in this study are available on request from the corresponding author. The data are not publicly available due to privacy concerns.
